# Bio-Fabrication of Human Amniotic Membrane Zinc Oxide Nanoparticles and the Wet/Dry HAM Dressing Membrane for Wound Healing

**DOI:** 10.3389/fbioe.2021.695710

**Published:** 2021-07-28

**Authors:** Palaniappan Ramasamy, Ramachandran Krishnakumar, Ravichandran Rekha, Baskaralingam Vaseeharan, K. Saraswathi, Mohan Raj, Robert E. B. Hanna, Gerard P. Brennan, Govindan Dayanithi, Sekar Vijayakumar

**Affiliations:** ^1^Research and Development Wing, Central Research Laboratory, Sree Balaji Medical College and Hospital, BIHER, Chennai, India; ^2^Cologenesis Healthcare Private Ltd., Salem, India; ^3^Department of Animal Health and Management, Alagappa University, Karaikudi, India; ^4^Department of Obstetrics and Gynaecology, Sree Balaji Medical College and Hospital, BIHER, Chennai, India; ^5^Department of Ophthalmology, Sree Balaji Medical College and Hospital, BIHER, Chennai, India; ^6^School of Biology and Biochemistry, The Queen’s University of Belfast, Belfast, United Kingdom; ^7^Veterinary Science Division, AgrI-Food and Biosciences Institute, Belfast, United Kingdom; ^8^Molecular Mechanisms in Neurodegenerative Diseases Laboratory, MMDN, University of Montpellier, L’École Pratique des Hautes Etudes-Sorbonne, INSERM, UMR-S1198, Montpellier Cedex 5, France; ^9^Marine College, Shandong University, Weihai, China

**Keywords:** amniotic membrane, wound healing dress, wet/dry membrane, proteins, biofabrication, HAMP’s-ZnO nanoparticles, antibacterial activities, biofilm

## Abstract

The preparation of unique wet and dry wound dressing products derived from unprocessed human amniotic membrane (UP-HAM) is described. The UP-HAM was decellularized, and the constituent proteins were cross-linked and stabilized before being trimmed and packed in sterile Nucril-coated laminated aluminium foil pouches with isopropyl alcohol to manufacture processed wet human amniotic membrane (PW-HAM). The dry type of PD-HAM was prepared by decellularizing the membrane, UV irradiating it, lyophilizing/freeze-drying it, sterilizing it, and storing it at room temperature. The UP-HAM consists of a translucent yellowish mass of flexible membranes with an average thickness of 42 μm. PW-HAM wound dressings that had been processed, decellularized, and dehydrated had a thinner average thickness of 30 μm and lacked nuclear-cellular structures. Following successful decellularization, discrete bundle of fibrous components in the stromal spongy layers, microvilli and reticular ridges were still evident on the surface of the processed HAM, possibly representing the location of the cells that had been removed by the decellularization process. Both wet and dry HAM wound dressings are durable, portable, have a shelf life of 3–5 years, and are available all year. A slice of HAM dressing costs 1.0 US$/cm^2^. Automation and large-scale HAM membrane preparation, as well as storage and transportation of the dressings, can all help to establish advanced technologies, improve the efficiency of membrane production, and reduce costs. Successful treatment of wounds to the cornea of the eye was achieved with the application of the HAM wound dressings. The HAM protein analysis revealed 360 μg proteins per gram of tissue, divided into three main fractions with MWs of 100 kDa, 70 kDa, and 14 kDa, as well as seven minor proteins, with the 14 kDa protein displaying antibacterial properties against human pathogenic bacteria. A wide range of antibacterial activity was observed after treatment with 75 μg/ml zinc oxide nanoparticles derived from human amniotic membrane proteins (HAMP-ZnO NP), including dose-dependent biofilm inhibition and inhibition of Gram-positive (*S. aureus, S. mutans, E. faecalis*, and *L. fusiformis*) and Gram-negative bacteria (*S. sonnei, P. aeruginosa, P. vulgaris, and C. freundii).*

## Graphical Abstract

Flow chart depicting the methods, preparing, and characterizing, by histological, and scanning electron microscopy, of wet (PW-HAM) and dry (PD-HAM)of wound healing dressing, and preparation of nanoparticles (HAMP ZnO NP); and application of HAM wound dressing.

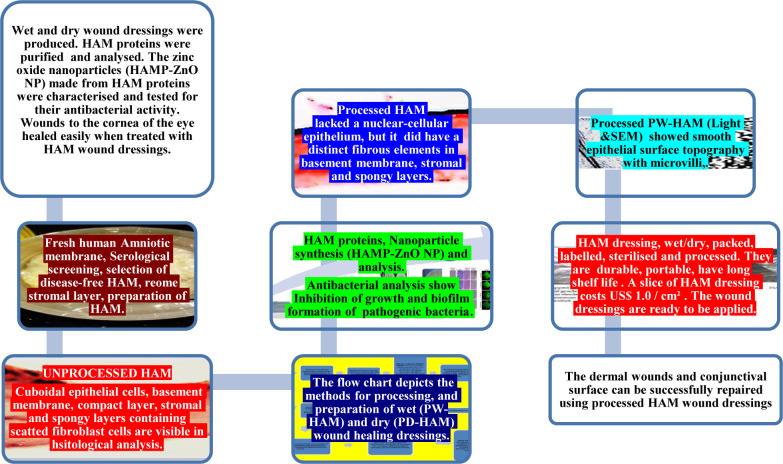

## Introduction

The human amniotic membrane (HAM), which consists of an epithelial layer, a basement membrane, and avascular stroma, is the human placenta’s innermost layer. The HAM can provide a pure, semipermeable membranous wound dressing that is non-immunogenic, reduces inflammation and pain, reduces scar tissue formation, and preserves all-natural wound healing properties after adequate processing. Antimicrobial properties, long-term release of a variety of growth factors and cytokines, and the strongest skin replacement for different wound applications are all features of these dressings. Furthermore, processed dressing (PW-HAM/DP-HAM) provides a favorable environment for cellular migration and dissemination, promoting faster healing and a wide range of wound-healing effects. Processed HAM is widely used in tissue engineering and plastic surgery, as well as in dermatology as a dressing for skin burns, diabetic and non-diabetic wounds, such as leg ulcers, and in ophthalmic healing (Davis, 1910; John, 2003; Dua et al., 2004; Gholipourmalekabadi et al., 2015). In addition to treating cutaneous wounds, HAM has a wide range of uses and can help restore different tissues (Meller et al., 2011; Saghizadeh et al., 2013; Mamede et al., 2015; Flügel et al., 2020). For protection, long-term storage, and successful application, appropriate procedures for handling and processing biological membranous structures such as HAM are needed. (i) hypothermic (fresh) storage at 4°C; (ii) cryo-preservation at −70°C; (iii) freeze-drying (lyophilization); and (iv) air-drying were used as preservation methods. Although these were the most popular preservation methods until recently, they have drawbacks and are impractical at certain times and in certain locations. Furthermore, it is thought that lyophilized HAM is best preserved with epithelial cells, which can be more immunogenic and thus cause rejection by reactive hosts. The immunogenic properties of lyophilized HAM can hinder the healing process, limiting its utility in promoting cell growth and tissue regeneration (Shortt et al., 2009). To address these drawbacks, HAM has been decellularized and stabilized using chemical or enzymatic methods, or a combination of both, to remove immunogenic cellular components while selectively stabilizing extracellular structural fibrous proteins. The biological properties of such a “decellularized scaffold membrane” have been demonstrated (Davis, 1910; Gipson and Grill, 1982; de Melo et al., 2007). Several methodologies for decellularizing and modifying the biomechanical and physico-chemical properties of the “HAM scaffold matrix” have been established in the past, but these methods only resulted in a compromised HAM product (Simon et al., 2003; Rieder et al., 2004). Bader et al. (2000) suggested a longer trypsin incubation time for more successful decellularization, but this disrupted the HAM matrix. To decellularize the HAM, a mixture of Tris buffer, protease inhibitors, nucleases, and Dispase II, as well as sodium dodecyl sulphate (SDS), was used (Wilshaw et al., 2008; Lim et al., 2009). The HAM had previously been treated with SDS, which had a destabilizing impact on the collagen matrix and induced swelling throughout the elastin framework (Samouillan et al., 1999). Dispase II treatment also caused significant changes in the structure of HAM’s basement membrane, rendering it unsuitable for explant culture development (Lim et al., 2009). Treatment with EDTA and/or proteolysis may also result in HAM damage (Saghizadeh et al., 2013; Sripriya and Kumar, 2016). Canciello et al. (2020) acknowledged that “An important goal that still remains to be achieved is the identification of cultural and preservation protocols able to maintain in time the membrane morphology and the biological properties of its cells.” It is critical to develop improved methods of preparation and application in order to address difficulties and limitations in the manufacturing, preservation, sterilization, and packaging methodologies, to maximize HAM protection and long-term storage, and to take advantage of the amniotic membrane’s potential benefits in a variety of applications. Therefore, the main object of the present investigation was to develop a novel process for preparing a sterile, stable, inexpensive human amniotic membrane product, free from the cellular elements of the chorion, for use as a wound dressing in dermal and ocular applications. This involved decellularization and cross linking of fibrous proteins, followed by stabilization, packaging and sterilization of HAM. Another aim of this study was to determine whether this prepared product could be used to treat such wounds topically. Antibacterial, antifungal, and anticancer properties have been found in a number of peptides, proteins, essential growth factors, and cytokines found in HAM. To broaden the therapeutic potential of HAM, a protein extract of amniotic membrane was combined with zinc oxide (ZnO) to form HAMP-ZnO nanoparticles (HAMP-ZnO NP), whose antibacterial activity was investigated.

## Materials and Methods

### Screening and Collection of HAM

**FIGURE 1:**
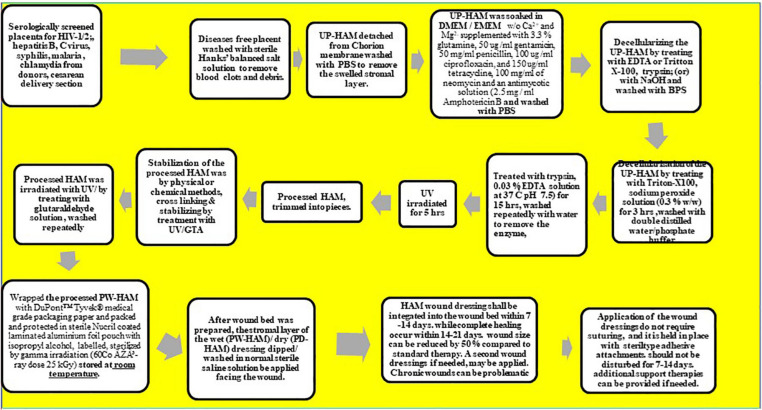
Flow chart shows the processes and preparation of HAM and application.

Fresh human placentae were obtained, with informed consent at the time of delivery, from mothers at the caesarean facility of the Department of Obstetrics and Gynecology, Sree Balaji Medical College and Hospital, Chennai, India. Disease-free placentae for HAM preparation were selected on the basis of serological screening for detectable pathogens viz. Hepatitis B and C viruses; HIV 1 and 2; syphilis, malaria, and *Chlamydia*. Human amniotic membrane was collected from the placentae in a sterile working area and washed with sterile Hanks’ balanced salt solution in a laminar air flow chamber to remove excess blood and blood clots. The material was washed with multiple changes of PBS, causing the stromal layer to swell and thus facilitating its removal. Further processing under sterile conditions followed the methods detailed in the flow chart (Figure 1) (Tseng et al., 1998; Rieder et al., 2004; Fareeha et al., 2010; Riau et al., 2010; Peter Crapo et al., 2011; Saghizadeh et al., 2013; Mafalda, 2015). In order to free the HAM from chorionic material and to cleanse it thoroughly from blood clots and cellular debris it was first soaked, then rinsed numerous times in Dulbecco’s Modified Eagle’s medium (DMEM) or in Eagles Essential Medium (EMEM). DMEM was supplemented with 3.3% glutamine, 4500 mg/L glucose, 110 mg/L sodium pyruvate and 4mg/L pyridoxine hydrochloride. EMEM contained Earle’s salts lacking Ca^2+^ and Mg^2+^, and was supplemented with 0.584 g/L glutamine and 2.2 gm/L sodium bicarbonate. Additionally, both DMEM and EMEM were supplemented with antibiotics (50 μg/mL gentamicin, 50 mg/mL penicillin, 100 μg/mL ciprofloxacin, 150 μg/mL tetracycline, 100 mg/mL of neomycin and 2.5 mg/mL of the antimycotic, Amphotericin B). Further the decellularization of the amniotic membrane was carried out by treating with EDTA (0.25% w/v), washing with 3% Triton X100 (0.3% w/v) in Tris-HCl buffer (10 mM, pH 8.0), washing with 0.1% EDTA at 4°C for 48 h, incubating with proteolytic enzyme (sterile 0.1% w/v trypsin), washing in 0.03% EDTA solution at 37°C, pH 7.5) for 15 h or with 0.5 M NaOH (for 10–30 s). This was followed by repeated washings with PBS and 0.02% EDTA for 24 h and with DMEM or EMEM which were supplemented with antibiotics (50 ug/ml gentamicin, 50 mg/ml penicillin, 100 μg/ml ciprofloxacin, 150 μg/ml tetracycline, 100 mg/ml of neomycin and 2.5 mg/ml of the antimycotic, Amphotericin B).

These processes are summarized in the flow chart (Figure 1). After spreading the decellularized AM on sterile nitrocellulose paper (GE Health care) it was cut into pieces of 1.5 × 1.5 cm^2^, 3 × 3 cm^2^, or 5 × 5 cm^2^, and stored at −20°C in small vials containing DMEM or EMEM without Ca^2+^ and Mg^2+^ until further use. A maximum of 15–17 HAM pieces of 5 cm size or 125–675 HAM pieces of 1 × 1 cm size were obtained from a single placenta. All investigations on HAM were carried out as approved by the Institutional Ethics Committee guidelines and regulations of Sree Balaji Medical College and Hospital, BIHER, Chennai, India.

### Wet Form of PW-HAM

The decellularized HAM was cross-linked and stabilized by treating with UV for 5 h or with a protein-protein cross-linker (1% glutaraldehyde) for 20 min, then washed with 1 M sodium cacodylate (pH 7.2) or 100 mM phosphate buffer (pH 7.5). The HAM was washed repeatedly for 4 h to get rid of the glutaraldehyde. Pieces of the amniotic membrane (1 × 1 cm^2^, 1.5 × 1.5 cm^2^, 3 × 3 cm^2^, 5 × 5 cm^2^) were cut and wrapped with DuPont^TM^ Tyvek^®^ medical grade packaging paper (Medical Packaging Knowledge Center^1^). They were then packed in sterile Nucril-coated laminated aluminum foil pouches with isopropyl alcohol, labeled and sterilized by gamma irradiation (^60^Co ray dose 25 kGy) (Genova et al., 1987; Branski et al., 2007; FerizAdrovic, 2012; ANSI/AAMI/ISO 11137-1:2006/(R), 2010) and the wet PW-HAM was stored in isopropyl alcohol for up to 5 years at 30°C before use.

### Dry Form of PD-HAM

The decellularized HAM was washed repeatedly with 100 mM phosphate buffer (pH 8.5). It was then irradiated with UV at 365 nm for 5 h or was cross-linked and stabilized by treating with 1% glutaraldehyde solution for 20 min. Following cross-linking with glutaraldehyde it was buffer-washed repeatedly for 4 h. The processed HAM was freeze-dried (lyophilized) by rapidly freezing the membrane, then reducing the pressure followed by heating to sublimate the water and so form a dry wound dressing (Kruse et al., 2000; Allen, et al., 2013). Freeze-dried PD-HAM was wrapped in DuPont^TM^ Tyvek^®^ medical packaging paper (Medical Packaging Knowledge Center^1^), then packed in sterile Nucril-coated laminated aluminum foil pouches, labeled and sterilized by gamma irradiation (^60^Co ray dose 25 kGy) (Genova et al., 1987; Branski et al., 2007; FerizAdrovic, 2012; ANSI/AAMI/ISO 11137-1:2006/(R), 2010). The packages were stored for up to 5 years at 30°C before use.

### Histological Analysis

Unprocessed and processed samples of HAM were fixed in neutral buffered formalin (10% v/v in 1M sodium cacodylate buffer pH 7.2 or sodium phosphate buffer pH 7.5), washed repeatedly to remove the fixative, dehydrated and embedded in paraffin wax. Three μm tissue sections were cut using a microtome, stained with Hematoxylin and Eosin (H&E) and examined with a Carl Zeiss Microscope. The differences between the unprocessed and processed HAM samples were analyzed (de Melo et al., 2007; Sanluis-Verdes et al., 2015).

### Scanning Electron Microscopy (SEM)

Processed and unprocessed HAM samples were fixed for 24 h in 4% glutaraldehyde (EM grade) prepared in 1 M sodium cacodylate buffer (pH 7.2) containing 3% sucrose and NaCl. After fixation the tissues were washed 5 times with the buffer, dehydrated in a graded series of ethanol (20 min in each), further dehydrated in acetone, critical point-dried, sputter-coated with gold palladium and finally examined in a scanning electron microscope (Zhang et al., 2016).

### Use of Wet (PW-HAM) or Dry (PD-HAM) Dressing for Conjunctival Surface Reconstruction

After the wound bed on the conjunctiva was prepared with the aid of topical anesthesia, or the defective pterygium of the eye was surgically removed, the wet (PW-HAM) or dry (PD-HAM) dressing was rinsed by dipping in normal sterile saline solution and then the new membrane was applied to the wound with the epithelial side facing outward and the membrane was secured in place by suturing. The saline-rinsed PW-HAM or PD-HAM was allowed to glide under the lower eyelid. The dressing was chosen to promote epithelialization and inhibit inflammation and angiogenesis. It was used for conjunctival surface reconstruction, in pterygium surgery and for curing other ocular surface problems (Prabhasawat et al., 1997; Espana et al., 2002; Sangwan et al., 2007).

### Extraction of Protein From Amniotic Membrane and Determination of Antibacterial Activities

The HAM was separated from the outer membrane of the embryo, washed in a phosphate buffer (pH 8.5) and stored on ice or at −20°C until use. The amniotic membrane (1800 gm) was homogenized, sonicated, and centrifuged at 10000 RPM for 15 min. The resulting protein-containing extract (HAMPE) was frozen at –80°C (Shao et al., 2004). Protein density was measured by using a nano-drop ND-1000 spectrophotometer, ThermoFisher Scientific, United States. The protein extract prepared from the UP-HAM was purified by using AKTA pure -Sephadex ion-exchange column chromatography (Wipro GE Healthcare Pvt Ltd, Life Sciences). The protein extract of the UP-HAM was subjected to SDS-PAGE protein analysis (Laemmli, 1970) in a discontinuous buffer system at pH 8.3. The UP-HAM protein sample was mixed with sample buffer (2X) in 1:1 ratio, heated in a boiling water bath for 5 min, loaded into the SDS-PAGE 10% as separating gel and 4% polyacrylamide as stacking gel and run at a constant voltage (60 V) for 6–8 h. After protein separation, the gel was incubated in 10% Trichloroacetic acid (TCA) for 60 min to fix the HAM proteins, stained with Coomassie brilliant blue/amido black for 6 h at room temperature followed by de-staining and the gels were stored in 7% acetic acid. The molecular weight of the HAM proteins was determined with molecular weight markers by using Gel Doc 2000 (BioRad) software. Antibacterial inhibitory effects of the HAMPE/purified proteins (HAMP’s) on the growth of bacteria were determined by adopting the well diffusion assay method of Bauer et al. (1966). The column-purified 14 kDa protein of UP-HAM was loaded (25, 50, and 75 μg protein/mL) into the wells of Mueller Hinton agar (MHA) plates, seeded with 20 μl of bacterial cultures of *Pseudomonas aeruginosa* (10^6^ cell/mL) incubated at 37°C for 24 h and the zone of inhibition was determined.

### Synthesis of HAMP-ZnO NP

Reduction of zinc acetate dehydrate to zinc oxide nanoparticles was carried out following the co-precipitation method of Singh et al. (2011) with slight changes. In brief, 2 M zinc acetate was prepared in 50 mL of deionized water with vigorous stirring. Five ml of HAMPE was added and the mixture was stirred continuously with a magnetic stirrer for 3 h. The resultant white precipitate was filtered and washed carefully with double distilled water to remove impurities. The white powder obtained was dried using a vacuum desiccator at 27 ± 3°C.

### Characterization of HAMP-ZnO NP

The HAMP-ZnO NP suspension (1.5 mL of aliquots) was sonicated for uniform dispersion, and evaluated by measuring the UV spectrum in a cuvette. The aqueous components were analyzed in the range 200–600 nm using an UV-Vis spectrophotometer (UV-1800; Shimadzu, Japan). A solution of zinc acetate (2 mM) was used as blank. The crystalline nature of HAMP -ZnO NPs was determined by X-ray diffraction (Powder X-ray diffractometer, X’ Pert Pro-P Analytic). The scanning was performed in the region of 2-THETA from 30° to 90°. For FTIR (Fourier Transform Infrared Spectroscopy) studies, HAMP-ZnO NPs (1 mg) were mixed separately with 100 mg of spectroscopic grade KBr in about 1:100 proportion. The mixture was then compressed into a 2 mm semi-transparent disk by applying a force of 10 T for 2 min. The FTIR spectra were recorded in the wavelength of 400–4000 cm^–1^ at a resolution of 4 cm^–1^ (PerkinElmer, Shelton, CT, United States).

### Antibacterial Activity of HAMP-ZnO NP (Well Diffusion Assay)

The antibacterial activity of HAMP-ZnO NP was evaluated against Gram positive bacteria (*Staphylococcus aureus, Streptococcus mutans, Enterococcus faecalis*, and *Lysinibacillus fusiformis*) and Gram-negative bacteria (*Shigella sonnei, Pseudomonas aeruginosa, Proteus vulgaris*, and *Citrobacter freundii*) by well diffusion assay. HAMP-ZnO NP (25, 50, and 75 μg/mL) were loaded into the wells of Mueller Hinton agar (MHA) plates seeded individually with 20 μl of one of the targeted bacterial cultures (10^6^ cell/mL). The plates in triplicates were kept in an incubator at 37°C for 24 h and the zone of inhibition around the wells indicating the antibacterial activity of HAMP’s -ZnO NPs was measured in diameter (mm) and were compared with control experiments. A zone of inhibition was determined both in controls [Zinc acetate (75 μg mL^–1^)] and in the HAMP-ZnO NPs (75 μg/mL) against harmful bacteria such as *E. faecalis, S. aureus, P. aeruginosa, P. vulgaris, S. mutans, S. sonnei, L. fusiformis*, and *C. freundii*.

### Antibiofilm Activity of HAMP-ZnO NP

The biofilm thickness of Gram positive (*S. aureus, S. mutans, E. faecalis*, and *L. fusiformis*) and Gram-negative bacteria (*S. sonnei, P. aeruginosa, P. vulgaris*, and *C. freundii*) and the antibiofilm efficacy of HAMP’s -ZnO NPs were evaluated through CLSM (Confocal Laser Scanning Microscopy) analysis. Quantitative assessment of biofilm inhibition in the presence of HAMP’s -ZnO NPs was performed in 24 well polystyrene plates. In brief, sterilized glass pieces were aseptically introduced into the wells of 24 well plates containing 20 μl of known cultures in 1. 5 mL of Luria Bertani broth supplemented with HAMP’s -ZnO NPs at 25, 50, and 75 μg/mL individually. Control was maintained with bacterial cultures alone and the plate was incubated for 24 h at 37°C. After incubation, the used media was gently removed from the 24 wells and the glass pieces were washed thrice with 0.1 M PBS to remove loosely attached bacterial cells and other polluting materials. The plate was air dried for 15 min and the glass pieces were stained with 0.4% acridine orange. Prior to visualization, the excess of stain was removed and air-dried. The images of the stained-glass pieces were observed under CLSM (LSM 710, Carl Zeiss, Zana, Germany) equipped with an excitation filter 515–560 and magnification at 20×. For light microscopy analysis, the glass pieces were stained with (0.1%) crystal violet and after air-drying were examined microscopically at 40 x magnifications.

## Results

### Light and Scanning Electron Microscopy of Unprocessed and Processed HAM

**FIGURE 2:**
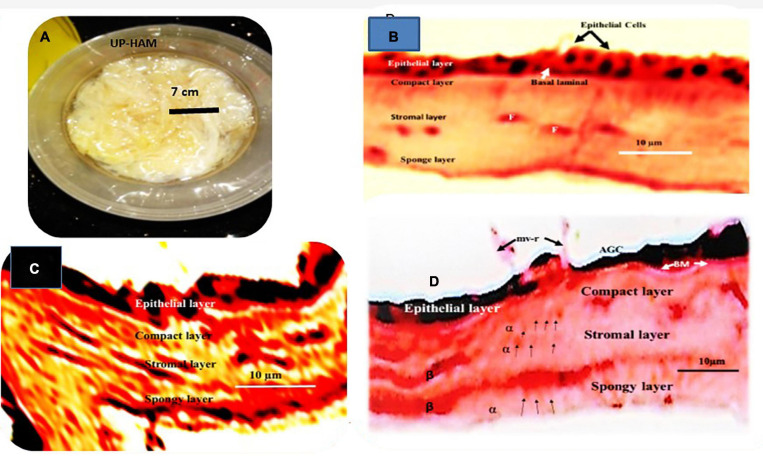
**(A)** A photograph depicts a bright yellowish tinted mass of fresh unprocessed human amniotic membranes (UP-HAM) that is slimy, flexible, and transparent. **(B)** A part of an unprocessed amniotic membrane (UP-HAM) stained with Hematoxylin and Eosin is shown in a light micrograph. There are cuboidal epithelial cells, a fibrous basement membrane, a stroma made up of a compact layer, a stromal layer made up of scatted fibroblast cells in a fibrous matrix, and a spongy layer. Hematoxylin is used to stain the nuclei of epithelial cells. Cells of the fibroblast (F). **(C)** Displays a cross-sectional view of the processed decellularized unique PD-HAM membrane stained with Hematoxylin and Eosin (H&E). In the decellularized, dehydrated, processed PD-HAM, there are bundles of fibrous components present in the compact, stromal, and spongy layers, as well as the absence of nuclear-cellular structures. With H&E, the epithelial layer is often stained black. **(D)** Displays a cross-sectional view of the processed decellularized PD-HAM membrane stained with Hematoxylin and Eosin (H&E). In the decellularized, dehydrated, processed DP-HAM, bundles of fibrous components (unique α and β long fibers) can be seen, as well as the absence of nuclear-cellular structures. Hematoxylin and Eosin is used to stain the decellularized epithelial layer. AGC stands for apical membrane glycocalyces, BM stands for basement membrane, and mv-r stands for microvillous ridge.

**FIGURE 3:**
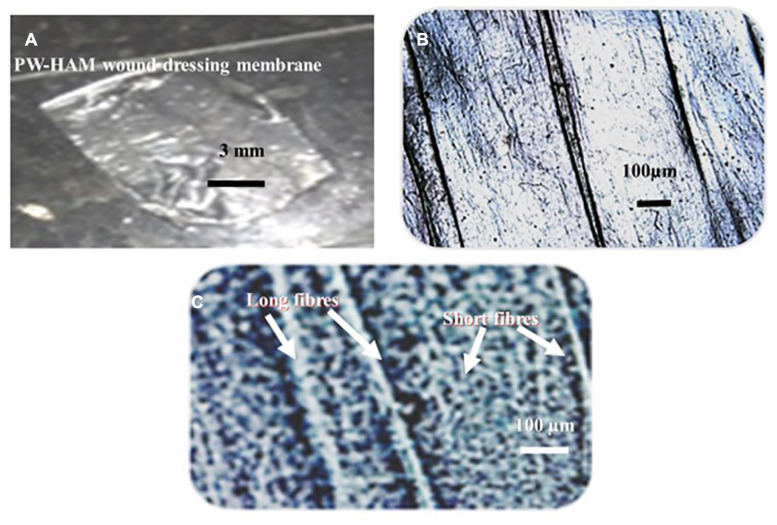
**(A)** A translucent PW-HAM wound dressing unpacked from the DuPont^TM^ Tyvek^®^ medical grade packaging paper and sterile Nucril coated laminated aluminum foil pouch. **(B)** A light micrograph showing a smooth surface view of the matrices of the processed PW-HAM wound dressing. **(C)** A light micrograph shows fibrous nature (unique long and short fibers) of the matrices of the processed PW-HAM wound dressing.

**FIGURE 4:**
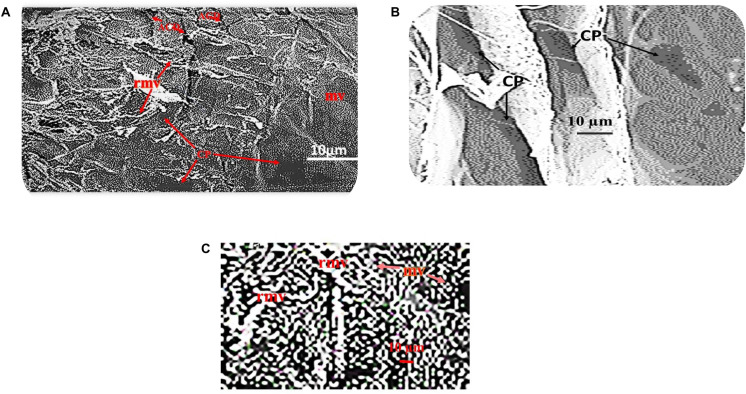
**(A)** SEM micrograph shows microvilli (mv) on the amniotic epithelial surface of the processed PW-HAM wound dressing. Surface area of some amniotic epithelial surface lacking microvilli shows depressions (ACD) with pit-like structures (CP) and reticular ridges (rr). **(B)** SEM micrograph of the amniotic epithelial surface of the processed PW-HAM wound dressing indicate the occurrence of microvilli. Some epithelial surface lacks microvilli but have pit-like depressions (CP). **(C)** Microvilli (mv) or reticular microvilli (rmv) of the amniotic epithelial surface of the processed PW-HAM wound dressing are visible in a magnified SEM micrograph.

Unprocessed HAM appears as a yellowish transparent mass of flexible membranes with an average thickness of 42 μm when removed from the chorion (Figures 2A,B). The epithelium is preserved as a single layer of cuboidal cells varying in thickness from 5 μm to 10 μm in samples freshly isolated and stored in 70% ethanol, underneath which is a dense acellular basement membrane composed of reticular fiber, preceded by a fibrous avascular stroma of connective tissues consisting of a compact layer, stromal and spongy layers (Figure 2B). Cuboidal epithelial cells, fibrous basement membrane, and a spongy stromal layer containing scatted fibroblast cells were visible in Hematoxylin and Eosin-stained portions of unprocessed HAM sheets (Figure 2B). Hematoxylin was used to dye the nuclei of cells in the epithelium and stromal layers, while Eosin was used to stain the cytoplasm (Figure 2B). The dried, decellularized, dehydrated HAM was thinner than the unprocessed HAM, varying in thickness from 20 μm to 40 μm with an average thickness of 30 μm and lacking a cellular epithelium (Figures 2C,D, Figures 3A,C). The fibrous basement membrane and a distinct bundle of fibrous components in the stromal spongy layers, such as thick longitudinal and thin fibers, persisted (Figures 2C,D, Figures 3B,C). Nuclear-cellular structures were missing from the dried, decellularized HAM membrane (Figures 2C,D). The decellularized, refined HAM wound dressing presented a smooth, fibrous, and glassy transparent appearance (Figures 3A–C). The processed PW-HAM dressing had a relatively smooth and flat surface topography with microvilli and reticular ridges on a fine matrix, as seen in SEM micrographs (Figures 4A–C). Some regions of the surface lacked microvilli and had pit-like depressions (Figures 4A,B), which might represent the locations of epithelial cells lost during the preparative processes. Cross-linking with glutaraldehyde solution for 20 min, followed by washing with buffers (1M sodium cacodylate buffer for 4 h) contributed to the biomembrane’s stability and retention of biological and wound healing properties. The cost of production of a slice of HAM dressing is 1.0 US$/cm^2^. The HAM wound dressing can be stored at room temperature (30°C) for 3–5 years, is portable, and is available throughout the year.

### Use of PW-HAM/PD-HAM for Conjunctival Surface Reconstruction

**FIGURE 5:**
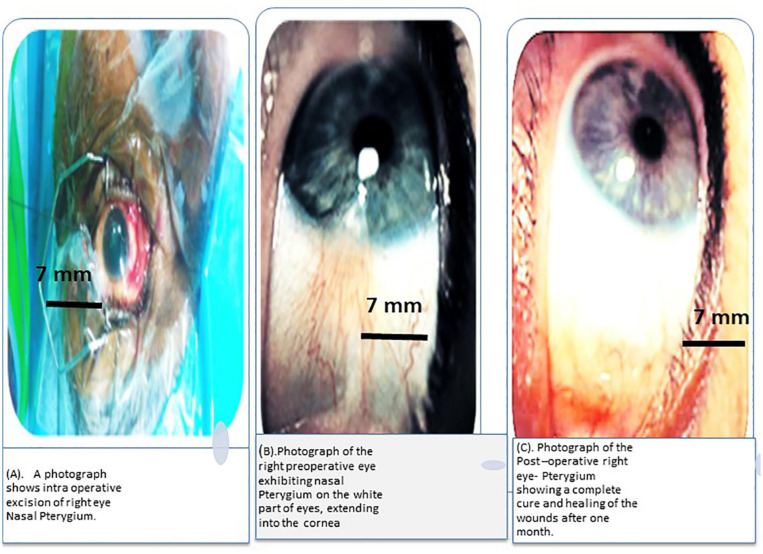
**(A)** A photograph depicts the removal of a Nasal Pterygium from the right eye during surgery. **(B)** Preoperative photograph of the right eye showing nasal Pterygium, a mucous membrane that protects the white portion of the eye and extends into the cornea. **(C)** After 1 month, the post-operative right eye-pterygium has fully healed and the wounds have healed completely.

The new membrane was found to be securely attached to the conjunctiva seven days after a processed PW-HAM/PD-HAM dressing was applied to the surgical wound caused by removal of a nasal pterygium. The development of a functional epithelium was complete after 21 days, and the applied membrane appeared to dissolve and vanish. Figures 5A–C depict the pre-operative and post-operative appearances of the eye, as well as the location of the processed HAM during surgery. This study found that processed human HAM membrane can be used to successfully repair the conjunctival surface after a nasal pterygium is surgically removed.

### Extraction of Protein From UP-HAM and Determination of Antibacterial Activities

**FIGURE 6:**
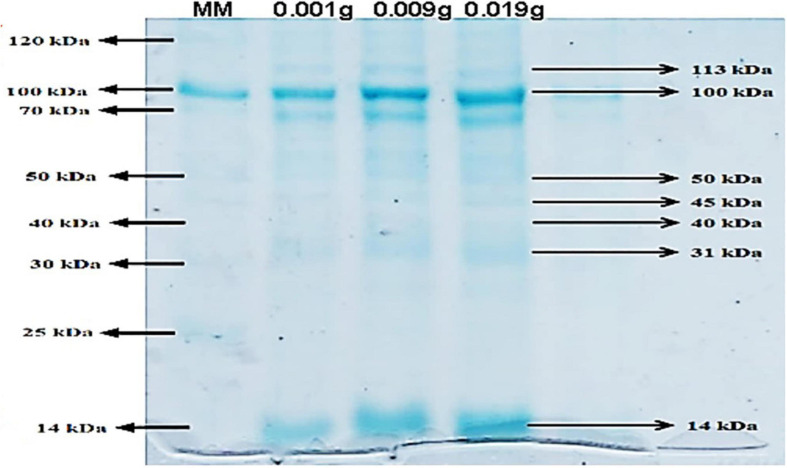
Purified Human amniotic membrane proteins (HAMPs) on SDS-PAGE with a 10% separating gel and a 4% polyacrylamide stacking gel. MM stands for protein molecular weight marker, and Lane II-IV stands for purified HAMPs (Note the presence of 14–113 kDa proteins).

**FIGURE 7:**
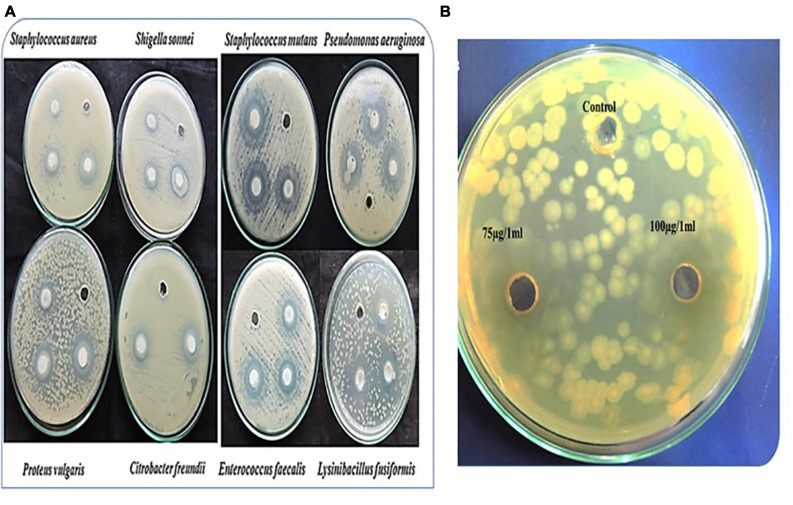
**(A)** depicts a zone of inhibition of HAMP-ZnO NPs against Gram positive (*Staphylococcus aureus, Streptococcus mutans, Enterococcus faecalis* and *Lysinibacillu, fusiformis*) and Gram-negative bacteria (*Shigella sonnei, Pseudomonas aeruginosa, Proteus vulgaris* and *Citrobacter freundii).* **(B)** shows a zone of inhibition of the 14 kDa protein of HAMPs against *Pseudomonas aeruginosa*.

The protein density of the UP-HAM extract was 360 μg of protein per gram of tissue. AKTA pure-Sephadex ion-exchange column chromatography was used to further purify the protein extract. PAGE analysis showed the existence of three prominent proteins, with molecular weight of 100, 70, and 14 kDa (Figure 6). In addition, the Coomassie Brilliant Blue-stained gels revealed the presence of six or seven other minor proteins. When applied to a culture plate of *Pseudomonas aeruginosa*, the 14 kDa purified protein of UP-HAM was found to have antibacterial activity, creating a zone of inhibition (Figures 7A,B). A zone of inhibition (in mm) was found both in controls Zinc acetate (75 μg/mL^–1^) and HAMP-ZnO NPs (75 μg/mL) against harmful bacteria such as *E. faecalis, S. aureus, P. aeruginosa, P. vulgaris, S. mutans, S. sonnei*, and *C. freundii*, albeit the zone of inhibition was much greater in HAMP-ZnO NPs (Figure 7A; see Tables 1, 2).

**TABLE 1:** The zone of inhibition (mm) of HAMP-ZnO NPs against pathogenic bacteria viz. *C. freundii, E. faecalis, L. fusiformis, P. aeruginosa, P. vulgaris S. aureus, S. mutans, S. sonnei* are detailed.

S. No.	Bacterial strains	Accession numbers	Concentrations of HAMP-ZnO NPs		
			25 μg/mL	50 μg/mL	75 μg/mL
			Zone of inhibition (mm)		
1	*Proteus vulgaris*	HQ116441	2.0 ± 0.3	3.0 ± 1.1	4.0 ± 1.1
2	*Pseudomonas aeruginosa*	HQ 4006631	3.0 ± 0.2	3.0 ± 1.2	4.0 ± 1.2
3	*Lysinibacillus fusiformis*	KF886273	1.3 ± 0.3	4.3 ± 1.1	4.3 ± 1.1
4	*Shigella sonnei*	ATCC-25175	1.3 ± 0.3	4.3 ± 1.1	4.3 ± 1.1
5	*Citrobacter freundii*	KC465905	2.0 ± 0.3	4.0 ± 1.1	5.0 ± 1.1
6	*Staphylococcus aureus*	HQ693279.1	3.1 ± 0.1	5.1 ± 0.4	5.1 ± 0.4
7	*Enterococcus faecalis*	MTCC-9542	3.0 ± 0.2	4.0 ± 0.4	6.0 ± 0.4
8	*Streptococcus mutans*	K7769531	2.0 ± 0.3	4.30 ± 1.1	7.0 ± 1.1

*The antimicrobials activity of the HAMPs was evaluated by the formation of a microbial zone of inhibition technique which showed a clear zone of inhibition (Figures 6A,B: Table 1). An increase in the zone of inhibition exhibited by the bacterial pathogens varied with increase in concentration of HAMPs. The maximum zone of inhibition exhibited by *S. mutans* was (7.0 ± 1.1 mm), followed by *E. faecalis* (6.0 ± 0.4 mm), *S. aureus* (5.1 ± 0.4 mm), *C. freundii* (5.0 ± 1.1 mm), *S. sonnei* and *L. fusiformis* (4.3 ± 1.1 mm), and P. vulgaris (4.0 ± 1.1 mm).*

**TABLE 2:** The inhibition zones (mm) of Zinc acetate (control) and HAMP-ZnO NPs against pathogenic bacteria are detailed.

S. No	Bacteria strains and accession numbers	Zinc acetate (75 μg mL^–1^)	Concentrations of HAMP-ZnO NPs 75 μg/mL
		Zone of inhibition (mm)	
1	*Streptococcus mutans* K7769531	2.8 ± 0.1	7.0 ± 1.1
2	*Enterococcus faecalis* (MTCC-9542)	3.1 ± 0.1	6.0 ± 0.4
3	*Staphylococcus aureus* (HQ693279.1)	3.0 ± 0.4	5.1 ± 0.4
4	*Pseudomonas aeruginosa* (HQ 4006631)	2.3 ± 0.1	4.0 ± 1.2
5	*Proteus vulgaris* (HQ116441)	3.3 ± 0.2	4.0 ± 1.1
6	*Shigella sonnei* (ATCC-25175)	2.0 ± 0.2	4.3 ± 1.1
7	*Citrobacter freundii* (KC465905)	2.6 ± 0.1	5.0 ± 1.1

*Both Zinc acetate and HAMP-ZnO NPs showed antimicrobial efficacy against pathogenic bacteria such as *E. faecalis, S. aureus, P. aeruginosa, P. vulgaris, S. mutans, S. sonnei*, and *C. freundii*. The zone of inhibition was greater in HAMP-ZnO NPs than in Zinc acetate in all bacterial species investigated, though it varies per bacterial pathogen.*

### Characterization of HAMP-ZnO NPs

**FIGURE 8:**
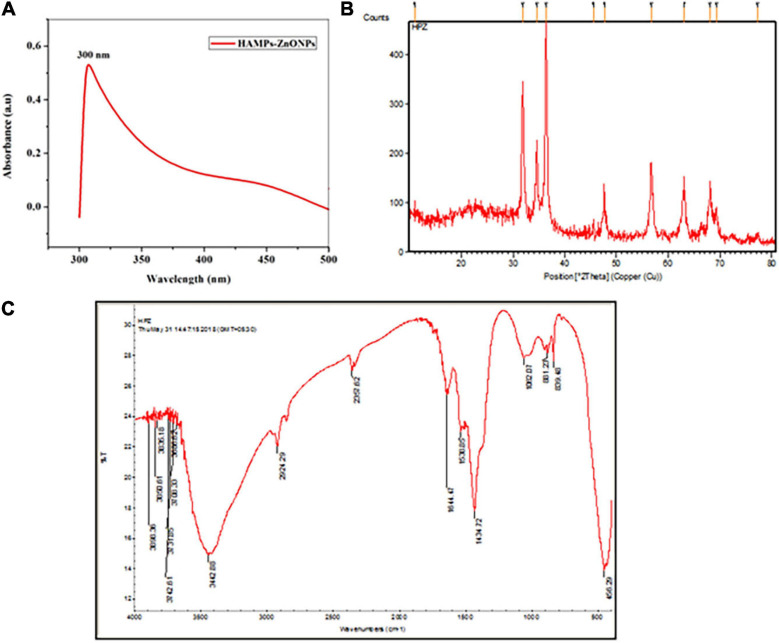
**(A)** In the synthesized HAMP’s-ZnO NPs, the UV Spectroscopy spectrum shows a sharp peak at 300 nm. **(B)** The reflection peaks of the synthesized HAMP Zinc oxide nanoparticles can be seen in the XRD spectrum. **(C)** The peaks of the functional groups of the synthesized Zinc oxide nanoparticles can be seen in the FTIR spectrum.

The synthesized HAMP-ZnO NPs had a sharp peak at 300 nm, according to UV spectroscopic analysis (Figure 8A). X-ray diffraction analysis confirmed the crystalline structure of HAMP-ZnO NPs (XRD). In the 2-THETA range between 10° and 80°, XRD analysis of HAMP-ZnO NPs revealed Bragg’s reflection peaks at 31.7, 34.3, 36.1, 47.4, 56.5, 62.7, and 69.9 (Figure 8B). These peaks were designated the diffraction lattice plates (100), (002), (101), (102), (110), (103), and (201), suggesting the existence of a crystalline structure with hexagonal wurtzite-shaped functional groups in HAMP-ZnO NPs. The XRD research findings match those recommended by the Joint Committee on Powder Diffraction Standards (JCPDS 75-0576). In the range of 400–4000 cm^–1^, the FTIR spectrum of HAMP-ZnO NPs was identified, revealing the existence of certain functional groups in the biosynthesized nanoparticles (Figure 8C). N-H stretching vibrations of amide groups in the protein lead to the band at 3442 cm^–1^. The peak at 2924 cm^–1^ is a C-H stretching vibration, while the peaks at 1644 cm^–1^ and 1434 cm^–1^ are carbonyl stretch and N-H stretch vibrations resulting from the protein’s amide II linkages. The existence of amine groups is indicated by the band at 1062 cm^–1^. The ZnO stretching mode is correlated with the band at 456 cm^–1^. These findings signify the active participation of the aforementioned functional groups in the HAM proteins present in HAMP -ZnO NPs.

### Antibacterial Potential of HAMP-ZnO NPs

HAMP-ZnO NPs demonstrated strong antibacterial activity against targeted bacteria as compared to control wells without the nanoparticle preparation (Figures 7A,B). Table 1 shows the diameters of inhibition zones for Gram positive and Gram-negative bacteria after exposure to 75 μg/mL of HAMP-ZnO NPs. HAMP-ZnO NPs is found to be effective against both Gram positive and Gram-negative bacteria. The 14 kDa protein adsorbed on the nanoparticles’ surface is thought to be responsible for HAMP-ZnO NPs’ antibacterial effectiveness.

### Antibiofilm Activity of HAMP-ZnO NPs

**FIGURE 9:**
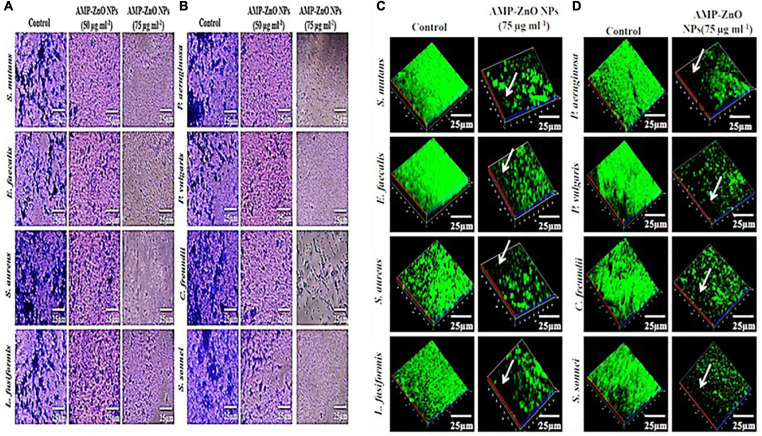
**(A,B)** Light micrographs show the antibiofilm activity of HAMP-ZnONPs (50 μg/ml and 75 μg/ml) against **(A)** Gram positive (*Staphylococcus aureus, Streptococcus mutans, Enterococcus faecalis* and *Lysinibacillus fusiformis*) and **(B)** Gram negative bacteria (*Shigella sonnei, Pseudomonas aeruginosa, Proteus vulgaris* and *Citrobacter freundii)*. The cells were stained with crystal violet. **(C,D)** Confocal laser scanning micrographs show the antibiofilm activity of HAMP-ZnO NPs (75 μg/ml) against **(C)** Gram positive (*Staphylococcus aureus, Streptococcus mutans, Enterococcus faecalis* and *Lysinibacillus fusiformis*) and **(D)** Gram negative bacteria (*Shigella sonnei, Pseudomonas aeruginosa, Proteus vulgaris*, and *Citrobacter freundii)*. The cells were stained with acridine orange.

Gram-positive (*S. aureus, S. mutans, E. faecalis*, and *L. fusiformis*) and Gram-negative bacteria (*S. sonnei, P. aeruginosa, P. vulgaris, and C. freundii*), both untreated controls and treated with HAMP -ZnO NPs, had their biofilm morphology assessed using light microscopy (crystal violet stain) and CLSM analysis (0.4% acridine orange nuclear stain). The findings are shown in Figures 9A–D. The biofilms treated with HAMP-ZnO NPs had a substantial reduction in thickness and coverage compared to the untreated control preparations, which showed complex multilayers of cells with good affinity to the substratum. With increasing concentrations of HAMP-ZnO NPs and incubation time, the inhibitory impact on biofilm thickness and coverage was stronger. As a result, HAMP-ZnO NPs inhibited biofilm formation in a dose-dependent manner.

## Discussion

The current study describes an updated processing and development procedure for stabilized, dry and wet formulations of a HAM wound dressing for topical application. These HAM wound dressings, both wet and dry, are inexpensive, readily available, easy to store and transport at room temperature, and can be used anywhere on the planet. Other similar preparations have been confirmed to produce inconsistent, ineffective, and substandard products, unlike the HAM wound dressings mentioned here. The preparation methods for HAM wound dressings, especially de-epithelialization, vary. EDTA, Trypsin-EDTA, Urea, Thermolysin, Ethanol, Hypotonic Buffer, SDS, and Nuclease have been used in this crucial step in the preparation (Jirsova and Jones, 2017; Ferenczy and Souza, 2020).

In the present report, a mixture of non-ionic detergents, alkali, and enzymatic treatments was used to fully decellularize the HAM while preserving the anatomical features of the basement membrane and avascular stroma. The decellularized and stabilized HAM dressing was found to be transparent, solid, and flexible, allowing it to conform to tissue contours in any anatomical position where it was applied as a wound dressing. The chelating agent EDTA aided metal ion sequestration and dissolution of immunogenic protein–protein linkages, resulting in epithelial cell disaggregation and dissociation from the HAM extracellular matrix (Aplin, 2003; Bacakova et al., 2004; Bhatia et al., 2007; Koley and Bard, 2010; Peter Crapo et al., 2011; Allen, et al., 2013; Fu et al., 2014; Mafalda, 2015; Sripriya and Kumar, 2016). After treating the HAM with the protein-digesting enzyme trypsin, peptide bonds were hydrolyzed and some of the complex proteins were broken down into smaller peptides. Triton-X100 disrupted hydrophobic polar head groups involved in hydrogen bonding in cellular lipid bilayers, allowing the HAM to be decellularized more easily. At low concentrations of TX100, the detergent monomer is known to be incorporated into lipid membranes, causing disruption and release of cellular contents. When cells are exposed to large quantities of TX100 for an extended period of time, the membrane permeabilization and structural failure are irreversible (Aplin, 2003; Bacakova et al., 2004; Bhatia et al., 2007; Koley and Bard, 2010; Allen, et al., 2013; Fu et al., 2014; Mafalda, 2015). Human amniotic membrane was previously treated with 1 percent Trypsin/EDTA, which eliminated epithelial and mesenchymal cells while keeping the basal membrane and extracellular matrix intact. The latter was mechanically powerful, had anti-inflammatory, anti-microbial, anti-fibrotic, anti-scarring, and biocompatibility properties, but had only a minor immunogenicity (Rieder et al., 2004; Young et al., 2005; Baguneid et al., 2006; Bhatia et al., 2007; Lim et al., 2009; Peter Crapo et al., 2011; Caruso et al., 2013; Mamede et al., 2015; Sanluis-Verdes et al., 2015; Zhang et al., 2016). The protection of essential molecules such as anti-inflammatory cytokines, growth factors, and extracellular matrix factors, as well as the structural integrity of the non-cellular matrix, should be the focus of future advances in the processing and stabilization of HAM for wound dressing. The results of the current study show that freeze drying HAM and preserving wet HAM wound dressings in isopropyl alcohol are the best methods for producing high-quality wound dressings. Furthermore, HAM wound dressings, whether wet or dry, are cost-effective, simple to collect, store, and transport at 30°C, and can be used anywhere in the world. Tseng and Tsubota’s (2002) procedures, on the other hand, involve freezing and storing pieces of HAM membrane in 50% glycerol in Dulbecco’s Modified Eagle Medium for up to 2 years, after which the membrane product must be thawed at room temperature and washed with normal buffered saline solution before use (Tseng et al., 1999; Tseng and Tsubota, 2002). In another procedure, the HAM membrane was first washed with dimethyl sulfoxide (DMSO) (0.5 M) 4% w/v in 0.01M phosphate-buffered saline solution (PBS), then MDMSO (8% w/v in 0.01 M PBS), and finally 1.5 M DMSO (12% w/v in 0.01 M PBS) (Tseng and Tsubota, 2002). As a result, the manufacturing and preparation methods for dry and wet HAM wound dressings, as well as the preservation and transportation conditions, differed significantly. In a number of climatic, economic, and geographic conditions, there is no standard operating procedure for various arrangements, storage, and transportation of HAM dressings. An automated universal process for production of HAM wound dressing preparations would improve the quality and quantity of the membrane for use, which could be crucial in assuaging users’ concerns and ensuring that the wound dressing is safe and widely accepted. Several layers were evident in both the unprocessed and processed HAMs using light microscopy and SEM. UP-HAM is a transparent yellowish mass of flexible membranes with an average thickness of 42 μm, according to the present study. Unlike UP-HAM, PW-HAM wound dressings were dried, decellularized, and dehydrated, with a thinner average thickness of 30 μm and no nuclear-cellular structures, appearing as a smooth, fibrous, and glassy translucent layer. An epithelial layer of cuboidal cells with hematoxylin-stained nuclei, as well as fibroblast cells in the stroma, were found in UP- HAM. Despite the fact that the decellularization process was effective because the processed HAM had no cells with stained nuclei, microvilli and reticular ridges were visible on a relatively smooth and flat amniotic surface. The current results are consistent with previous findings on unprocessed HAM, which revealed a layer of cuboidal epithelial cells, a fibrous basement membrane layer, a stromal fibrous layer with occasional fibroblast cells, and a spongy layered system. A high concentration of hydrated proteoglycans and glycoproteins, as well as type I and III collagen, was identified in the spongy sheet. Anti-angiogenic and anti-inflammatory proteins were present in the AM’s epithelial and mesenchymal cells, on the other side (Koizumi et al., 2000; Rieder et al., 2004, 2006; Hopkinson et al., 2006; Wilshaw et al., 2008; Liang et al., 2009; Peter Crapo et al., 2011). The denudation protocol used in this study involved removing cuboidal amniotic epithelial cells and other cells with a detergent, sodium dodecyl sulphate (SDS). More research is required to better understand the process of “synthetic HAM membrane preparation” and to establish better methods for completely extracting immunogenic components while preserving the stromal components’ molecular integrity. The cost of manufacturing a slice of HAM dressing was 1.0 US$/cm^2^, according to the actual figures derived from the current study. The Z-AM-3 × 3 dry amniotic membrane (3 cm × 3 cm) from Zabbys cost $100.00. (Cost of Dry Amniotic Membrane – Zabbys). The cost of amniotic membranes, on the other hand, ranged from $300 to 900 per device, putting a strain on patients who are paying out of pocket (Gutiérrez-Moreno et al., 2011; Voigt and Anderson, 2019: Voigt, 2020). Automation and large-scale HAM membrane preparation, as well as storage and transportation of the dressings, can all help to establish advanced technologies, improve the efficiency of membrane production, and reduce costs. Moreover, the HAM wound dressing can be stored for 3–5 years at room temperature (30°C), is compact, and is available all year.

The protein content of the UP-HAM was revealed to be 360 μg proteins/gm of HAM tissue in the current research. Three prominent proteins with molecular weights of 100 kDa, 70 kDa, and 14 kDa, as well as seven minor proteins, were discovered using SDS-PAGE. One of these purified proteins (the 14 kDa component) was found to have antibacterial activity against human pathogenic bacteria, indicating that HAM wound dressing could be used to prevent bacterial infection. Several other proteins contained in amniotic membrane extract have been shown to have antimicrobial and other functions (Yadav et al., 2017; Ramuta et al., 2020). Hopkinson et al. (2006) found that thrombospondin, mimecan, BIG-H3, integrin alpha 6 extracellular matrix-associated, and cell structural proteins had a substantially lower protein content. On fresh UP-HAM and samples taken at every stage of the processing and preparation, 2D analysis and protein microarray studies can help explain protein dynamics, including molecular and structural changes. The efficient synthesis and use of Zinc oxide HAMP-ZnO NPs using protein extract from the unprocessed human amniotic membrane was demonstrated in the current study. Bragg’s reflection peaks were identified in the HAMP-ZnO NPs after XRD analysis. The study revealed a crystalline structure with functional groups in a hexagonal wurtzite form. The observations of the present study were in agreement with previous findings on zinc-oxide nanoparticles (Le-ZnO-NPs) and CS/Ag/ZnO nanocomposite (Thaya et al., 2016; Vijayakumar et al., 2019). Purification, peptide and NP synthesis, as well as characterization and antimicrobial testing of protein fragments isolated from human amniotic membrane stem cells, are all important requirements in the establishment of innovative antibacterial molecules and applications.

The HAMP-ZnO NPs were also found to have significant antibacterial activity against Gram positive and Gram-negative bacteria. The concentration of HAMP-ZnO NPs was found to increase the clear zone of inhibition, a measure of antimicrobial activity. Furthermore, the inhibition zone for Gram positive bacteria was larger than that for Gram negative bacteria (see Table 1). Naskar et al. (2020) also found that AZO NPs had lower antibacterial activity against Gram-negative bacterial cells *E. coli* and *A. baumannii* than *S. aureus*, despite MRSA3 and MRSA6 being more resistant. Despite the fact that antibacterial activity of ZnO NPs and Zn^2+^ ions has been reported, the exact mechanism of antibacterial activity is unknown. ZnO NPs and Zn^2+^ ions are thought to inhibit genomic DNA/plasmid replication and influence the development of bacterial cell membrane proteins/enzymes, resulting in cell death. The ability of the synthesized HAMP-ZnO NPs to kill Gram-positive and Gram-negative bacteria demonstrates the preparation’s broad antibacterial capacity, but further *in vitro* and *in vivo* research is needed to determine their full potential for infection control.

The anti-biofilm potential of HAMP-ZnO NPs was found to be proportional to both the concentration and the length of incubation with the nanoparticles. The thickness of the biofilms for both Gram positive and Gram-negative bacteria was decreased after treatment with nanoparticles. In the absence of the HAMP-ZnO NPs, the control cultures revealed highly complex multilayered biofilms with a strong affinity for the substrate. When Gram positive bacteria *(Bacillus licheniformis and Bacillus cereus)* and Gram-negative bacteria *(Bacillus niger)* were treated with CS/Ag/ZnO nanocomposite at 8 μg mL^–1^ they were found to have antibacterial activity and repress biofilm formation *(Vibrio parahaemolyticus, and Proteus vulgaris). Candida albicans* biofilm formation was also inhibited by the CS/Ag/ZnO nanocomposite at 50 μg mL^–1^ (92%) (Thaya et al., 2016; Vijayakumar et al., 2019). Jain et al. (2020) successfully synthesized ZnO nanoparticles that had excellent antimicrobial activity and applicability against *Xanthomonas oryzae pv. oryzae* and *Alternaria* sp. Green silver nanoparticles in a hybrid biological nano-scaffold cross-linked HAM, as well as the extract and Human Amnion Membrane Composites, improved antibacterial properties and wound healing, as well as their biomedical applications (Ramesh et al., 2017; Yadav et al., 2017; Palanker et al., 2019; Dadkhah Tehrani et al., 2021). Therefore it can be concluded that HAMP-ZnO nanoparticles (NPs) containing human amniotic membrane protein may have antimicrobial activity against a wide range of Gram positive and Gram negative bacterial pathogens that both impede and promote wound healing. More research into the production of new zinc oxide nanoparticles based on HAM proteins and related proteins, growth factors, and hormones, as well as their inhibitory effects, survival and growth of pathogenic bacteria in humans, could pave the way for more efficient non-antibiotic treatments and wound healing methods and technologies.

## Conclusion

The production and development of a wet (PW-HAM)/dry (PD-HAM) human amniotic membrane wound dressing for topical use with, in particular, dermal and ocular applications has been described in the current study. After decellularizing the amniotic membrane and cross-linking and stabilizing the proteins with UV/1%glutaraldehyde solution for 20 min, the wet form of the HAM dressings was made by packing them in 3 mL isopropyl alcohol, sterilizing them, and keeping them at room temperature (30°C) until required. Decellularizing the membrane, UV irradiating it, lyophilizing/freeze-drying it, sterilizing it, and storing it at room temperature created the dry form of HAM. The UP-HAM is a transparent yellowish mass of flexible membranes with a 42 μm average thickness. Processed, decellularized, and dehydrated PW-HAM wound dressings had a thinner average thickness of 30 μm and lacked nuclear-cellular structures. Following the decellularization process, a fibrous basement membrane, discrete bundle of fibrous components in the stromal spongy layers, microvilli and reticular ridges remained on a relatively smooth and flat membrane surface, over a fine matrix. The HAM wound dressings can be kept at room temperature for 3-5 years, are easy to transport, and are available all year. The cost of producing a slice of HAM dressing is low (1.0 US$/cm^2^). Automation and large-scale HAM membrane preparation, as well as the development of synthetic membranes, storage, and transportation of dressings, can all help to develop advanced technologies, increase membrane production quality, and lower costs. For a long time, HAM has been used in surgical reconstruction and tissue engineering. The HAM protein analysis indicated 360 μg proteins per gram of tissue, with three main fractions with MWs of 100 kDa, 70 kDa, and 14 kDa, and seven minor proteins. The 14 kDa protein has antibacterial properties against human pathogenic bacteria. Antibacterial activity was observed in HAMP-ZnO nanoparticles (NPs) containing protein isolated from the human amniotic membrane against Gram positive and Gram-negative bacteria. Furthermore, HAMP-ZnO NPs’ anti-biofilm ability was likely found to be proportional to incubation concentration. Treatment with HAMP-ZnO NPs inhibited and reduced the thickness of biofilm formation in Gram positive (*S. aureus, S. mutans, E. faecalis*, and *L. fusiformis*) and Gram-negative bacteria (*S. sonnei, P. aeruginosa, P. vulgaris*, and *C. freundii*). Innovative zinc oxide nanoparticles based on HAM antimicrobial proteins/peptides, growth factors, and signaling molecules, metabolites, as well as their synergistic effects on human bacterial infections, could pave the way for more successful wound healing methods and technologies, as well as antibacterial treatment molecules.

## Data Availability Statement

The raw data supporting the conclusions of this article will be made available by the authors, without undue reservation.

## Ethics Statement

The studies involving human participants were reviewed and approved by the Institutional Ethics Committee of Sree Balaji Medical College and Hospital (Reg. ID: IEC/CT/SBMCH/003/2015). The patients/participants provided their written informed consent to participate in this study.

## Author Contributions

PR initiated and performed the experiments, and drafted the main manuscript. Overall planning, guidance, and writing of the work including SEM work were carried out by GB, RH, and GD. HAM was processed, decellularized, cross-linked, stabilized, freeze-dried, packed, labeled, and irradiated, and both wet and dry HAM were prepared by PR and RK. KS carried out the cesarian operation, screened and selected diseases free placental tissues, and detached fresh amniotic membrane at the time of delivery in the Department of OBG of the hospital, Sree Balaji Medical College and Hospital, Chennai, India. MR performed conjunctival surface reconstruction and Pterygium surgery, and cured ocular surface. BV, RR, and PR performed protein separation, purification, nanoparticles synthesis, characterization, and antibiotic inhibitory effects of the proteins of the amniotic membrane. SV performed the formal analysis. All the authors provided inputs for the manuscript writing and discussion.

## Conflict of Interest

RK was employed by company Cologenesis Healthcare Pvt. Ltd. The remaining authors declare that the research was conducted in the absence of any commercial or financial relationships that could be construed as a potential conflict of interest.

## Publisher’s Note

All claims expressed in this article are solely those of the authors and do not necessarily represent those of their affiliated organizations, or those of the publisher, the editors and the reviewers. Any product that may be evaluated in this article, or claim that may be made by its manufacturer, is not guaranteed or endorsed by the publisher.

## Footnotes

1
www.dupont.co.in
